# A Case-Control Study to Investigate the Serotypes of *S. suis* Isolates by Multiplex PCR in Nursery Pigs in Ontario, Canada

**DOI:** 10.3390/pathogens9010044

**Published:** 2020-01-05

**Authors:** Leann C. Denich, Abdolvahab Farzan, Robert Friendship, Emily Arndt, Marcelo Gottschalk, Zvonimir Poljak

**Affiliations:** 1 Department of Population Medicine, University of Guelph, 50 Stone Rd E, Guelph, ON N1G 2W1, Canada; afarzan@uoguelph.ca (A.F.); rfriends@ovc.uoguelph.ca (R.F.); arndte@uoguelph.ca (E.A.); zpoljak@uoguelph.ca (Z.P.); 2 The Research Group on Infectious Diseases in Production Animals, University of Montreal, 3200 Sicotte, Saint-Hyacinthe, Montréal, QC J2S 2M2, Canada; marcelo.gottschalk@umontreal.ca

**Keywords:** swine, *Streptococcus suis*, serotypes, systemic and non-systemic sites

## Abstract

*Streptococcus suis* naturally inhabits the tonsils and nasal cavities of pigs. Some strains can cause systemic infection, leading to a wide range of diseases. A case-control study was conducted to (i) examine serotypes isolated from systemic sites (blood/meninges/spleen) in cases, (ii) determine whether serotypes in systemic sites were found in upper respiratory sites (tonsil/nasal cavity) of the same cases, and (iii) determine the serotypes in upper respiratory sites of case and farm and pen- matched controls. In total, 606 samples from 128 pigs were cultured for *S. suis*. The isolates were examined for presence of *gdh* and *recN* genes by polymerase chain reaction (PCR) and were identified as *S. suis* if both genes were present. The *S. suis* isolates were then serotyped using a two step-multiplex PCR. Serotypes 9 (n = 9), (2,1/2) (n = 7) and untypable isolates (n = 7) were most commonly found in systemic sites. Detection of serotypes 9 (*p* = 0.03) in upper respiratory sites were positively associated with their detection in systemic sites of cases, while a trend was seen with serotype (2,1/2) (*p* = 0.07). Last, no association between serotypes recovered from upper respiratory sites of cases and controls could be detected. Untypable isolates were detected in high frequency, which warrants further investigation. This study confirms that a variety of serotypes can be found in commercial swine production and shows a difference in serotypes recovered from systemic sites in pigs with clinical signs of *S. suis* infections.

## 1. Background

*Streptococcus suis* infections have become a major problem in the swine industry worldwide [1]. This bacterium is a Gram-positive facultative anaerobe that naturally inhabits the tonsils and nasal cavities of pigs, with most pigs remaining healthy carriers. Outbreaks tend to be sporadic and generally involve only a small number of at-risk pigs. Typically, the age group at greatest risk of developing clinical signs of infection are four- to eight-week-old piglets, and systemic infection in these pigs can result in a wide range of disease conditions [2]. In addition, *S. suis* is potentially zoonotic, with people who work closely with infected pigs or pork-derived products being at greatest risk in western countries [3]. Worldwide, there has been over 1000 *S. suis* human cases and over 100 deaths since 1968, with the most cases occurring in Vietnam and Thailand through the ingestion of contaminated raw pork products [4,5].

Twenty-nine known serotypes have previously been identified based on the capsular polysaccharide (cps) [4]. Most *S. suis* isolates recovered from diseased pigs belong to serotypes 1 through 9, 1/2 and 14, though the distribution may differ depending on geographical location [5,6]. In Canada, the most frequent serotypes recorded in diseased pigs (in decreasing order) are serotypes, namely 2, 3, 1/2, 8, 4, and 7 [4,7,8]. There are also untypable strains, which may be novel serotypes or mutants of known serotypes [1].

The most commonly recognized clinical expression of *S. suis* infection include neurological clinical signs, including ataxia, incoordination, tremor/shaking, paddling, opisthotonos, paralysis, convulsions, nystagmus, and often sudden death [6]. Disease tends to have a low incidence (<5%) on most farms. Factors related to host (immunity, upper respiratory diseases, microbiome, and genetics), environment (farm management, stocking density, ventilation, and temperature) and bacteria (virulence associated factors) likely play a role in the development of clinical illness. However, there are knowledge gaps with respect to why some animals become systemically ill, while most remain healthy, and how the bacterium spreads among pigs in a specific population. Both uncertainties add additional challenges when attempting to determine what triggers some pigs to become sick. A pig-level, farm- and pen-matched case-control study was conducted with the overarching goal to understand factors contributing to *S. suis* disease status in nursery pigs.

The objectives of this study were to: (i) examine *S. suis* serotypes isolated from systemic infections in sick pigs in Ontario, Canada, (ii) investigate whether serotypes found in systemic sites of sick pigs were also found in the upper respiratory sites of the same pigs, and (iii) compare the serotypes found in upper respiratory sites of sick and healthy pigs.

## 2. Results

### 2.1. Streptococcus suis Serotypes Recovered from All Samples

In total, 128 pigs (64 healthy pigs and 64 pigs with clinical signs of *S. suis* infection) on 12 farms were sampled. A total of 606 samples were collected, of which 359 were from clinically ill (case) pigs and 247 were from healthy (control) pigs. In total, 310 *S. suis* isolates (215 from cases and 95 from controls) were recovered. *Streptococcus suis* was isolated from at least one sample in 114 pigs (60 cases and 54 controls). At the pig level, *S. suis* was recovered most frequently from the tonsillar swabs in 69.4% of cases and 58.7% of controls (Table 1). The multiplex PCR was able to determine the serotypes of 178 typable isolates (122 from cases and 56 from controls), while 132 isolates (74 from cases and 58 from controls) remained untypable.

**Figure 1:**
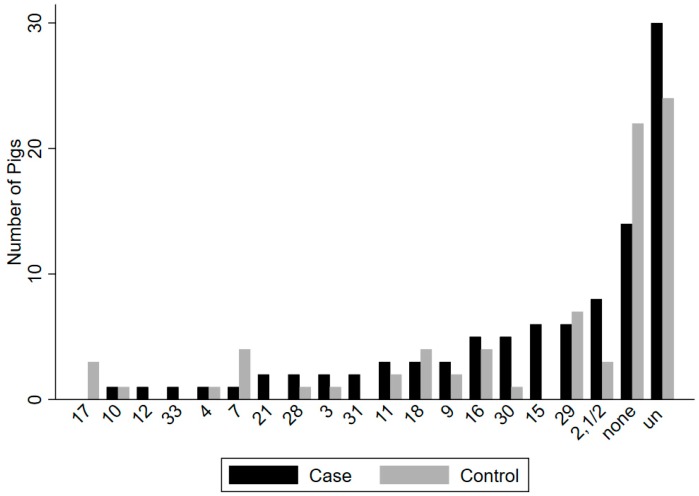
Comparison of the number of *Streptococcus suis* serotypes detected in upper respiratory (tonsil/nasal cavity) sites in 47 of 64 case animals and in 38 of 64 control animals on 12 Ontario farms. The most frequent serotypes in case animals included (2,1/2) (n = 8), 15 (n = 6), 16 (n = 5), 29 (n = 6), and 30 (n = 5), along with untypable (un) isolates (n = 30). The most frequent serotypes in control animals included 29 (n = 7), 9 (n = 3), 7 (n = 4), 16 (n = 4), and 18 (n = 4), along with untypable isolates (n = 24).

**Figure 2:**
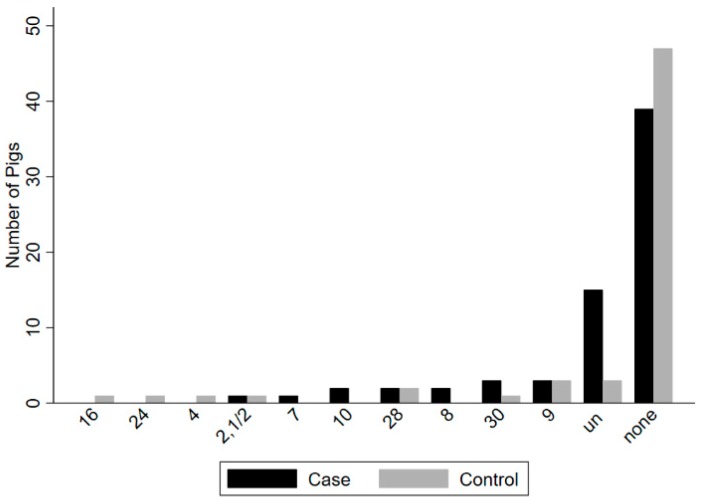
Comparison of the number of *Streptococcus suis* serotypes detected from rectal swabs in 25 of 64 clinical case animals and in nine of 64 control animals on 12 Ontario farms. The most frequent serotypes in case animals included 9 (n = 3), 30 (n = 3) and untypable (un) isolates (n = 15). The most frequent serotypes in control animals included serotype 9 (n = 3) and 28 (n = 2), as well as untypable isolates (n = 3).

Seventeen known serotypes and untypable isolates were recovered from upper respiratory (tonsil/nasal cavity) sites in 47 of 64 case and in 38 of 64 control animals (Figure 1), and 10 known serotypes and untypable isolates were recovered from rectal sites in 22 of 64 case and in 10 of 64 control animals (Figure 2). Serotypes recovered most frequently from case animals in upper respiratory sites (tonsil/nasal cavity) included (2,1/2) (n = 8), 15 (n = 6), 29 (n = 6), 16 (n = 5), and 30 (n = 5), as well as untypable isolates (n = 30), while the most frequent serotypes recovered from the rectum of case animals included 9 (n = 3) and 30 (n = 3), as well as untypable isolates (n = 15). In control animals, the most frequent serotypes recovered in upper respiratory sites (tonsil/nasal cavity) included 29 (n = 7), 7 (n = 4), 16 (n = 4), 18 (n = 4), and 9 (n = 3), as well as untypable isolates (n = 24), while serotypes most frequently recovered from the rectum of control animals included serotypes 9 (n = 3), 28 (n = 2), and untypable isolates (n = 3).

When considering all sample types, a single serotype was found in 12 case animals and 18 control animals, while two or more serotypes were recovered in 47 case animals and 22 control animals. With respect to the population of clinical cases, tonsillar swabs had the greatest number of different serotypes recovered (n = 13), followed by nasal cavity swabs (n = 12) and meningeal swabs (n = 11). Similarly, in the population of controls, tonsillar swabs also had the greatest number of different serotypes recovered (n = 12), followed by nasal cavity swabs (n = 8) and rectal swabs (n = 7).

**Figure 3:**
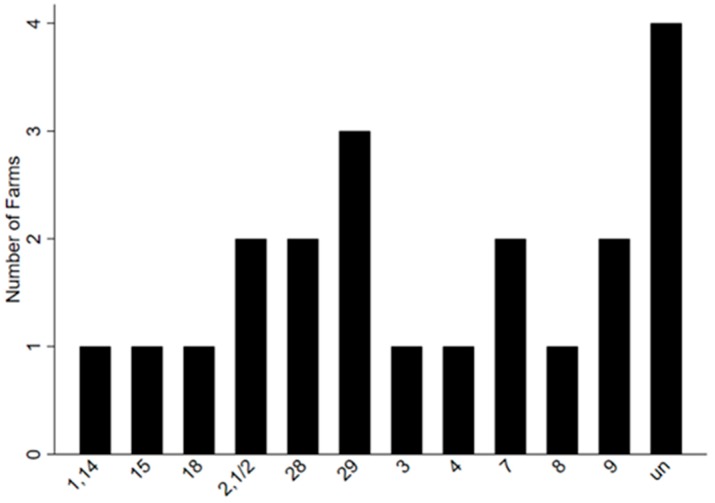
Number of *Streptococcus suis* serotypes detected on 12 farms in systemic (blood, meninges, spleen) sites of 28 confirmed cases with clinical signs of *S. suis* infections. The serotypes identified from most farms were 29 (n = 3), 7 (n = 2), 9 (n = 2), 28 (n = 2), and (2, 1/2) (n = 2), as well as untypable (un) isolates (n = 4).

The number of different serotypes found in 64 sampled clinical case pigs recovered on 12 farms can be seen in Figure 3 and the different serotypes found on each farm can be seen in Table 2. The number of serotypes detected at the level of the individual farm were between one and 15, with an average of seven.

### 2.2. S. suis Serotypes Recovered from Systemic Sites

The *S. suis* serotypes recovered from systemic sites of confirmed cases are shown in Table 3. The most commonly detected serotypes found in systemic sites at the pig level were 9 (n = 9), (2,1/2) (n = 7), and untypable isolates (n = 7). The most commonly detected serotypes found in systemic sites at the farm level included 29 on three farms, (2,1/2) and nine each on two farms, as well as untypable isolates on four farms.

Seventeen confirmed cases (61%) were identified with a single serotype in systemic sites, as well as nine cases (32%) with two serotypes, and two cases (7%) with three serotypes. The most common pattern of positivity for serotypes was positivity for serotype 9 only (n = 6), followed by positivity for serotype (2,1/2) only (n = 5) (Table 3). Furthermore, the most common pattern of positivity seen on farms in confirmed cases was detection of serotype (2, 1/2) (n = 2 farms) (Table 3).

### 2.3. Serotypes from Systemic Sites (Blood, Meninges and Spleen) Compared to Serotypes in Upper Respiratory Sites (Tonsils and Nasal Cavity) of Case Pigs

*Streptococcus suis* serotypes were not recovered from upper respiratory sites in seven confirmed (25%) cases. Similarly, a single serotype was also identified in seven confirmed cases (25%), as well as two serotypes in 10 (35.71%) confirmed cases, three serotypes (10.71%) in three confirmed cases, and five serotypes (3.57%) in one confirmed case. The same serotypes were recovered from systemic and upper respiratory sites in six (21.4%) confirmed cases. This included (2,1/2) (n = 2), 9 (n = 2), 18 (n = 1), and 31 (n = 1).

When univariable logistic regression was performed, there was an interest in how untypable isolates and serotypes 9 and (2, 1/2) compared between systemic and upper respiratory sites in confirmed cases (Table 4). Untypable isolates (n = 5) were frequently recovered in pigs in both sites, although there was no association between their detection in systemic and upper respiratory sites. Conversely, the presence of serotype 9 (OR = 5.6, *p* = 0.03) in upper respiratory sites were positively associated with the detection of the same serotype in the systemic sites of the same pig, while a similar positive trend was seen for serotype (2,1/2) (OR = 7.2, *p* = 0.07). However, it should be noted that isolation of serotypes found in both systemic and upper respiratory sites was low. This was also reflected in the low estimates of population attributable fraction for both serotypes (Table 4).

### 2.4. S. suis Serotypes in Upper Respiratory Sites (Tonsils and Nasal Cavity) of Case and Control Pigs

Together, 40 different serotype patterns were found in upper respiratory sites of 59 case and 59 matched control animals. The most frequent patterns included only untypable isolates in case (n = 12) and control (n = 18) animals, along with no *S. suis* recovered in case (n = 13) and control (n = 15) animals. In the subset of animals used for this analysis, zero to four serotypes were detected in upper respiratory sites of case and control animals. The most frequent serotypes in case animals included (2,1/2) (n = 8), 15 (n = 6), 9 (n = 5), 29 (n = 5), 30 (n = 5), and 16 (n = 4), along with 21 isolates which remained untypable. The most frequent serotypes in control animals included 29 (n = 6), 7 (n = 3), 16 (n = 4), 17 (n = 3), and 18 (n = 3), along with 29 isolates which remained untypable.

Seven matched case-control pairs (11.8%) shared at least one of the same serotypes in upper respiratory sites and can be seen in Table 5. These included serotype (2,1/2) from two farms (n = 3), serotype 18 from one farm (n = 2), serotype 30 from one farm (n = 1), and serotype 16 from one farm (n = 1). Untypable isolates were also present on eight farms. Univariable conditional logistic regressions showed there was no statistical evidence to suggest that the presence of a serotype in controls was associated with its presence in cases (Table 5).

## 3. Discussion

Diseases caused by *Streptococcus suis* are some of the most common bacterial diseases in the nursery stage of swine production. With a high frequency of healthy pigs carrying *S. suis*, there is a knowledge gap with respect to why some pigs become sick while others remain unaffected.

According to previous studies, the major serotypes of diseased pigs in North America have been 2, 3, and 1/2 [8,9]. There are some differences between these previously reported data and the present study. However, serotype (2, 1/2) was the most frequently isolated serotype found in clinical cases. Serotype 3 appeared in both case and control animals but was not found as frequently as the other serotypes. Interestingly, serotype 9, which, in previous studies has rarely been isolated from diseased pigs in Canada, appeared as frequently as serotype (2, 1/2) and its emergence has been shown in recent years [10].

In the present study, three different types of confirmed cases could be identified from descriptive statistics of positivity to different serotypes. First, there were *S. suis* cases which identified primarily with serotypes 9 and (2, 1/2) alone or in combination with other serotypes. The identification of clusters of cases within individual farms would be most consistent with either the clonal spread of one or more linkages of the same serotype among pigs, or more generally to the same source of infection [2,11]. Consistent with our research, a recent study in Germany also found systemic infections associated with serotype (2, 1/2) and serotype 9 [12]. Second, there was a subset of confirmed cases that were all detected with untypable isolates, alone or in combination with other serotypes. More discriminatory molecular typing is required in an attempt to understand the incidence of such confirmed cases. In the previous literature, untypable isolates were hypothesized to be defective mutants of strains of known serotypes or represent novel serotypes [8]. Last, there were confirmed cases that were detected with only one serotype, other than serotypes 9 or (2, 1/2). Such cases were observed in low frequency, with a maximum of two detections of serotype 3 on one farm. The low number of investigated clinical cases on some farms could have influenced this result and our interpretation. However, such detection is most consistent with existence of sporadic cases associated with different serotypes. These cases may be due to coinfections that have enabled the bacterium to reach systemic sites of these pigs [13].

Previous studies have also shown that *S. suis* serotypes recovered from systemic sites were generally different compared to those found in upper respiratory sites [14,15]. In the present study, only 21% of confirmed cases had serotypes in systemic sites that were the same to those in upper respiratory sites of the same pigs. When individual serotypes were evaluated, positive association between detection in upper respiratory sites and detection in systemic sites could be found only for serotypes 9 and a trend for serotype (2, 1/2). However, even for these two serotypes, only a small fraction of cases due to a specific serotype could be linked with detection of the same serotype in upper respiratory sites. Since multiple serotypes can colonize pigs, it is possible that our sampling and testing strategy resulted in low sensitivity of detecting a specific serotype in upper respiratory sites. However, this result also suggests that, when evaluated using a typical sampling and testing protocol, serotypes found in upper respiratory sites are not necessarily the same serotypes found in systemic sites causing pigs to become sick.

Further, there was no association between the presence of a serotype in upper respiratory sites of case and control animals. This suggests that we could not identify evidence that pen-mates serve as the source of colonization for cases, or that there is a common source of colonization for the majority of serotypes. Nonetheless, a combination of study design, small sample size and, possibly, low sensitivity of detecting a specific serotype in upper respiratory sites could also have negatively affected our ability to detect such associations.

This study has several limitations. It is possible that the clinically ill pigs were treated with an antibiotic prior to sampling, and as a result, some bacteria could have been eliminated from the blood and/or meninges and would therefore only be present in upper respiratory sites [16]. For the purpose of this study, we considered a combination of results from tonsillar and nasal swabs to be representative of the upper respiratory tract. It should be noted that not all sections of the upper respiratory tract could be sampled. Multiple serotypes were also found in systemic sites and could be suggestive of infection with more than one serotype and/or contamination. Consistent with previous observations reported, the use of the two-step multiplex PCR was also not able to distinguish between serotypes 1 and 14 and 2 and 1/2 [17,18]. Since we used PCR typing, we could have used the term capsular types as a surrogate for serotypes, but we decided to use serotypes in order to be consistent with the literature. Additionally, only one healthy animal was tested for each sick animal. Therefore, the diversity of serotypes at the farm level may have been underestimated. Last, it is also possible that issues with processing of samples such as transporting, storing, and culturing could have resulted in false negative results. Nevertheless, as many samples were obtained from both sick and healthy pigs, a snapshot of the current situation of *S. suis* in Ontario nurseries can be seen.

## 4. Conclusions

In conclusion, most commonly detected serotypes in systemic infections in *S. suis* diseased pigs in this study were serotypes (2,1/2) and 9, followed by frequent isolation of PCR untypable isolates from systemic sites. The colonization of upper respiratory sites with serotype 9 was positively associated with detection of these serotypes in systemic sites while a trend was seen for serotype (2, 1/2). However, only a small fraction of detection in systemic sites could be linked with the detection of the same serotype in upper respiratory sites (up to 20%, as indicated by the estimate of the population attribution fraction), making this result of limited practical use. Last, no link between the colonization of upper respiratory sites of case and control animals could be detected. Further research on the *S. suis* whole genome needs to be conducted to distinguish molecular differences of isolates. The high detection frequency of untypable isolates warrants further investigation.

## 5. Methods

### 5.1. Study Design and Sampling

A sample size calculation was based on visual inspection of power curves that were generated for a matched case-control study design using a power of 80%, confidence of 95%, and a ratio of cases and controls of 1. The prevalence of exposure in the control group was assumed to vary between a minimum of 0.12 and a maximum of 0.3. Under such assumptions, the number of cases and controls required to detect an odds ratio of 3 varied between 58 (control group exposure of 0.12) and 97 (control group exposure of 0.3). The required sample size was 31 cases if the magnitude of the odds ratio was 4. For field sampling, we targeted a minimum of 60 clinical cases and matched controls due to expected between farm variability and anticipated exclusion of clinical cases due to inadequate detection of *S. suis* in the sampling sites.

Owners of 12 farms located in Ontario, Canada, expressed interest in participating in this study because nursery pigs raised on these farms were experiencing typical clinical signs of *S. suis* infection.

Pigs showing clinical signs of *S. suis* infection (cases) were selected on each farm. For a pig to be considered a clinical case, they needed to exhibit at least one of the following clinical signs: Ataxia, incoordination, tremor/shaking, paddling, opisthotonos, paralysis, convulsions, and/or nystagmus. Each case was matched with an equal number of healthy pigs (controls) on each herd based on the time of visit and pen. For a pig to be considered a control, they needed to exhibit healthy and normal behavior, described as the absence of any of the above listed clinical signs.

Each case and control animal had one nostril, its tonsils, and its rectum swabbed, and blood sampled. In addition, cases were sedated with an intramuscular injection of a combination of ketamine (50 mg/mL), xylazine (10 mg/mL), and butorphanol (1 mg/mL) with a dosage of 0.2 mL/kg body weight and then euthanized with a 3-mL intracardiac injection of pentobarbital sodium (240 mg/mL). The skull was opened to collect meningeal swabs, and tissue samples were collected from the spleen. Samples were placed in a cooler after collection on the farm and brought to the laboratory located at the Centre for Public Health and Zoonoses (CPHAZ) at the University of Guelph for analysis. Samples were in the cooler for between 20 min and 2 h depending on the distance travelled to the farm.

### 5.2. S. suis Isolation and Identification

#### 5.2.1. *S. suis* Culturing

Tissue samples were placed in a paper boat and cut with a sterilized scalpel. Spleen samples, blood, and swabs (meninges, tonsil, nasal cavity, rectal) were plated on phenylethyl alcohol (PEA) agar and incubated at 35 °C with 5% CO_2_ for 48 h. One to four suspected *S. suis* colonies were selected per sample and plated on PEA agar and incubated at 35 °C with 5% CO_2_ for 48 h.

#### 5.2.2. DNA Extraction

Multiple suspected colonies were selected from PEA and DNA was extracted from isolates using InstaGene Matrix (Bio-Rad, Hercules, CA, USA) according to the manufacturer’s instructions. DNA samples were stored at −20 °C.

#### 5.2.3. *S. suis* Identification

To identify *S. suis*, DNA extracted from all selected suspected colonies were tested for the presence of the glutamate dehydrogenase (*gdh)* gene [19] and the recombination protein N (*recN)* gene by PCR [20]. The isolates were confirmed as *S. suis* if both genes were present. The confirmed *S. suis* isolates were then stored in CryoStor microbiology culture reservation vials according to the manufacturer’s instructions (BioLife Solutions, Bothell, WA, USA) for further identification.

#### 5.2.4. Serotyping

The *S. suis* isolates were analyzed using a two-step multiplex PCR method as described previously [18]. Capsular polysaccharide types determined by the PCR were examined by electrophoresis on a 1.5% agarose gel and visualized with RedSafe Nucleic Acid Staining Solution (FroggaBio, Toronto, ON, Canada).

#### 5.2.5. Categorization of Cases

Case pigs were divided into two major groups, including 28 confirmed cases and 32 probable cases. A pig was classified as a “confirmed case” if the pig displayed clinical signs at the time of visit and had the presence of *S. suis* recovered in systemic sites, including the blood, meninges, and/or spleen. A pig was classified as a “probable case” if the pig displayed clinical signs at the time of visit and had the presence of *S. suis* only recovered in any of the other locations sampled (tonsils, nasal cavity, or rectum). Four cases did not have *S. suis* detected from any site.

### 5.3. Statistical Analysis

Data were entered into a spreadsheet (Microsoft Excel 2016), cleaned, and then imported into Stata 15 (StataCorp, College Station, Texas, TX, USA) for further processing, descriptive statistics, and visualization. Descriptive statistics were conducted at the isolate level, pig level, and farm level. The isolate-level dataset was aggregated to a pig-level dataset, with individual variables representing the presence of *S. suis* to specific sampling sites for specific serotypes in individual pigs. Additional summary statistics were used to describe the detection of serotypes in upper respiratory sites and from rectal swabs in all available clinical case and control animals.

Objective 1. Twenty-eight confirmed cases were used to address Objective 1. Patterns of positivity were assessed by aggregating the number of confirmed cases detected with a specific serotype and with a combination of specific serotypes.

Objective 2. To investigate systemic and upper respiratory sites, the same 28 confirmed cases were used. A series of univariable exact logistic regression models was applied to investigate associations between the presence of a specific serotype in upper respiratory sites as a risk factor for detection of the same serotype in systemic sites of the same confirmed case. Analysis was performed only for serotypes that had at least three cases identified, and estimates were reported if *p* < 0.10. Population attributable fraction was determined using contingency tables.

Objective 3. To investigate the upper respiratory sites, only matched pairs that had sampling from both tonsil and nasal swabs were included for this objective. This resulted in a total of 59 of 64 clinically ill pigs and 59 of 64 individually matched healthy pigs that fit these criteria. A series of univariable conditional logistic regression models were used to investigate the association between the presence of a specific serotype in upper respiratory sites of clinically ill and healthy animals. Estimates were reported if *p* < 0.10.

## Figures and Tables

**Table 1:** The number of samples collected and tested positive for *S. suis* from case (sick with *S. suis* clinical signs) and control (healthy) animals at the pig level.

Sample-Type	Pig Level					
	Cases			Controls		
	Pigs Sampled (n)	Pigs Tested Positive (n)	% Pigs Positive	Pigs Sampled (n)	Pigs Tested Positive (n)	% Pigs Positive
Blood	62	9	14.5	62	0	0
Meningeal swabs	64	25	39.1	n/a	n/a	n/a
Spleen tissue	48	5	10.4	n/a	n/a	n/a
Tonsillar swabs	62	43	69.4	63	37	58.7
Nasal cavity swabs	60	27	45.0	64	17	26.6
Rectal swabs	63	22	34.9	58	10	17.2
Total	64	60	93.7	64	54	84.4

**Table 2:** *Streptococcus suis* serotypes found in case and control animals on each study farm based off all sampling sites.

Farm	Health Status	1, 14	2, 1/2	3	4	5	7	8	9	10	11	12	15	16	17	18	21	24	28	29	30	31	33	34	un
1	Case								+				+	+								+			+
	Control												+	+						+					+
2	Case																								
	Control																					+			
3	Case												+				+			+					+
	Control						+								+					+					+
4	Case		+	+			+	+					+							+	+	+			+
	Control						+							+	+					+		+		+	+
6	Case								+		+			+							+				+
	Control													+						+	+				+
7	Case		+		+	+	+	+		+	+		+	+		+			+	+	+	+			+
	Control		+				+				+					+				+	+				+
8	Case		+						+										+						+
	Control				+			+									+								+
9	Case																								+
	Control																	+							+
10	Case		+		+				+	+			+			+			+	+				+	+
	Control			+			+		+	+	+			+					+						+
11	Case	+	+	+						+	+	+		+					+			+			+
	Control		+	+							+														+
12	Case		+						+		+		+						+						+
	Control										+		+							+					+
13	Case				+	+		+	+					+											+
	Control				+				+					+						+					+
Total farms (n)		1	6	3	4	2	4	4	6	3	5	1	6	7	2	2	2	1	5	8	3	5	1	2	11

+: Present; un: Untypable.

**Table 3:** Pattern positivity for different serotypes found in systemic sites (blood, meninges, spleen) of 28 confirmed cases of *Streptococcus suis* infections at the pig and farm level.

Pattern id#	Pigs (n)	Farms (n)	Serotype (n)	un	1, 14	2, 1/2	3	4	7	8	9	15	18	28	29	31
1	1	1	1	-	-	-	-	-	-	-	-	-	-	-	-	+
2	1	1	1	-	-	-	-	-	-	-	-	-	+	-	-	-
3	6	1	1	-	-	-	-	-	-	-	+	-	-	-	-	-
4	1	1	3	-	-	-	-	-	-	-	+	-	-	+	+	-
5	1	1	2	-	-	-	-	-	-	-	+	+	-	-	-	-
6	1	1	1	-	-	-	-	-	+	-	-	-	-	-	-	-
7	2	1	1	-	-	-	-	+	-	-	-	-	-	-	-	-
8	1	1	1	-	-	-	+	-	-	-	-	-	-	-	-	-
9	5	2	1	-	-	+	-	-	-	-	-	-	-	-	-	-
10	1	1	2	-	-	+	-	-	-	-	-	-	+	-	-	-
11	1	1	3	-	-	+	-	-	-	-	-	-	+	-	+	-
12	1	1	1	-	+	-	-	-	-	-	-	-	-	-	-	-
13	2	1	1	+	-	-	-	-	-	-	-	-	-	-	-	-
14	1	1	2	+	-	-	-	-	-	-	-	-	-	-	+	-
15	1	1	2	+	-	-	-	-	-	-	+	-	-	-	-	-
16	1	1	2	+	-	-	-	-	-	+	-	-	-	-	-	-
17	1	1	2	+	+	-	-	-	-	-	-	-	-	-	-	-
Pigs (n)	28	n/a		6	2	7	1	2	2	1	9	1	3	1	3	1
Farms (n)	n/a	8		4	1	2	1	1	2	1	2	1	1	1	2	1

+: Positive; -: Negative; un: Untypable.

**Table 4:** Serotypes found in systemic (blood, meninges, and spleen) and upper respiratory (tonsil and nasal cavity) sites of 28 confirmed *S. suis* cases.

Serotype	Upper Resp ^a^ (+)		Upper Resp (−)		OR ^c^	PAF ^d^	CI ^e^	P ^f^
	Sys ^b^ (+)	Sys (−)	Sys (+)	Sys (−)				
1,14	0	0	2	26	Np ^g^	np	np	np
2,1/2	2	1	5	20	7.24	0.20	0.319, 494.7	0.07
3	0	1	1	26	np	np	np	np
4	1	0	2	25	-	-	-	0.78
7	0	0	2	26	np	np	np	np
8	0	0	1	27	np	np	np	np
9	2	0	7	19	5.59	0.16	0.4151, +inf	0.03
10	0	1	0	27	np	np	np	np
11	0	2	0	26	np	np	np	np
15	1	2	0	25	np	np	np	np
16	0	0	4	24	np	np	np	np
18	1	1	1	25	np	np	np	np
28	0	0	1	27	np	np	np	-
29	0	1	3	24	-	-	-	0.72
31	1	2	0	25	np	np	np	np
Un ^h^	4	12	1	11	-	-	-	0.25

^a^ Upper resp: upper respiratory sites; ^b^ sys: Systemic sites; +: Positive; -: Negative; OR ^c^: Odds ratio; PAF ^d^: Population attributable fraction; CI ^e^: Confidence interval; p ^f^: *p*-value. Note that confidence intervals and *p*-values were calculated using two different methods. Np ^g^ = Not performed; un ^h^: Untypable.

**Table 5:** *Streptococcus suis* serotypes found in upper respiratory (tonsil and nasal cavity) sites of 59 clinically ill and 59 healthy pigs.

Serotype	Clinically Ill Pigs (n)	Healthy Pigs (n)	(n) Matched Pairs ((n) on Farms)	P ^c^
2,1/2	7	4	3 (2 farms)	0.124
3	3	1	1 (1 farm)	Np ^d^
4	1	1	-	1
7	1	3	-	0.21
9	5	2	-	0.27
10	2	1	-	0.57
11	3	2	-	0.66
12	1	0	-	1
15	6	0	-	np
16	4	4	1 (1 farm)	1
17	0	3	-	np
18	3	3	2 (1 farm)	1
21	2	0	-	np
28	2	2	-	1
29	5	6	-	0.566
30	5	0	-	np
31	2	1	-	0.341
33	1	0	-	np
Un ^e^	21	29	15 (8 farms)	0.301

p ^c^: *p*-value. Np ^d^: Not performed; un ^e^: Untypable.

## Abbreviations

DNA: deoxyribonucleic acid; *gdh*: glutamate dehydrogenase; PCR: polymerase chain reaction; PEA: phenylethyl alcohol agar; *recN*: recombination protein N; *S. suis*: *Streptococcus suis*.
